# Does ethnicity affect pain management for people with advanced disease? A mixed methods cross-national systematic review of ‘very high’ Human Development Index English-speaking countries

**DOI:** 10.1186/s12904-022-00923-6

**Published:** 2022-04-06

**Authors:** Gemma Clarke, Emma Chapman, Jodie Crooks, Jonathan Koffman, Shenaz Ahmed, Michael I. Bennett

**Affiliations:** 1 Academic Unit of Palliative Care, Leeds Institute of Health Sciences, University of Leeds, Leeds, England UK; 2 Department of Palliative Care, Policy and Rehabilitation, Cicely Saunders Institute, King’s College London, London, England UK; 3 Division of Psychological & Social Medicine, Leeds Institute of Health Sciences, University of Leeds, Leeds, England UK

**Keywords:** Ethnicity, Race, Pain, Pain management, Health inequalities, Advanced disease, Palliative care, Systematic review, Mixed methods

## Abstract

**Background:**

Racial disparities in pain management have been observed in the USA since the 1990s in settings such as the emergency department and oncology. However, the palliative care context is not well described, and little research has focused outside of the USA or on advanced disease. This review takes a cross-national approach to exploring pain management in advanced disease for people of different racial and ethnic groups.

**Methods:**

Mixed methods systematic review. The primary outcome measure was differences in receiving pain medication between people from different racial and ethnic groups. Five electronic databases were searched. Two researchers independently assessed quality using JBI checklists, weighted evidence, and extracted data. The quantitative findings on the primary outcome measure were cross-tabulated, and a thematic analysis was undertaken on the mixed methods studies. Themes were formulated into a conceptual/thematic matrix. Patient representatives from UK ethnically diverse groups were consulted. PRISMA 2020 guidelines were followed.

**Results:**

Eighteen papers were included in the primary outcome analysis. Three papers were rated ‘High’ weight of evidence, and 17/18 (94%) were based in the USA. Ten of the eighteen (56%) found no significant difference in the pain medication received between people of different ethnic groups. Forty-six papers were included in the mixed methods synthesis; 41/46 (89%) were based in the USA. Key themes: Patients from different ethnically diverse groups had concerns about tolerance, addiction and side effects. The evidence also showed: cultural and social doctor-patient communication issues; many patients with unmet pain management needs; differences in pain assessment by racial group, and two studies found racial and ethnic stereotyping.

**Conclusions:**

There was not enough high quality evidence to draw a conclusion on differences in receiving pain medication for people with advanced disease from different racial and ethnic groups. The mixed methods findings showed commonalities in fears about pain medication side effects, tolerance and addiction across diverse ethnic groups. However, these fears may have different foundations and are differently prioritised according to culture, faith, educational and social factors. There is a need to develop culturally competent pain management to address doctor-patient communication issues and patients’ pain management concerns.

**Trial registration:**

PROSPERO-CRD42020167890.

**Supplementary Information:**

The online version contains supplementary material available at 10.1186/s12904-022-00923-6.

## Introduction

### Race, ethnicity and pain management

Pain can be a distressing and debilitating symptom. For people with advanced disease, prevalence data indicates that pain affects approximately 66% of those with advanced cancer [1]; 30–79% of those with end-stage liver disease [2]; 32–66% of those with Chronic Obstructive Pulmonary Disease (COPD) [3, 4]; and an estimated 46–56% of people with dementia [5]. Controlling pain is one of the central goals of palliative care. The World Health Organization (WHO) defines palliative care as “an approach that improves the quality of life of patients and their families facing the problems associated with life-threatening illness, through the prevention and relief of suffering by means of early identification and impeccable assessment and treatment of pain and other problems.” [6] Pain relief is also a fundamental human right [7, 8], and yet it remains a challenge globally. Undertreatment is common, despite effective treatments being available [9–11].

Pain is a multifaceted phenomenon. It can be related to social and cultural factors, as well as neurological and biological responses [12]. The biopsychosocial model of pain recognises the social and psychological dynamics of expressing pain [13]. and because pain experience is subjective, it can be particularly susceptible to these factors [14]. Societally disadvantaged groups may face greater barriers to pain management, for example: socioeconomic factors, gender, culture and ethnic background have all been shown to impact pain management [15–19]. Barriers to pain management can occur at a patient, physician, health service or community level, as wider societal disparities are replicated within healthcare through processes of discrimination, prejudice and structural impediments [20–22]. For people who are members of multiple disadvantaged groups, these barriers may interact in sequential, additive or intersectional ways to create greater and further disparities [23].

Racial and ethnic inequalities within healthcare have long been recognised internationally [24–27]. However recent events of historical and political significance, such as: the Black Lives Matter (BLM) movement [28]; Empire Windrush in the UK [29]; and the disproportionate impact of COVID-19 on people from Black and Asian groups [30–33], have drawn new attention to long standing issues. Pain management is one area with well documented and enduring disparities [15]. In the 1990s, research in the USA revealed that those from non-White groups were less likely to receive adequate analgesia in the emergency department [19], and in oncology settings [34]. More recently, a 2016 study demonstrated that a substantial number of White laypeople and medical students held false beliefs about biological differences between Black people and White people (e.g. “Black people have thicker skin”) [35]. In palliative care however, issues of race and ethnicity in pain management are not well described, and very few studies have examined race and racism.

### Previous systematic reviews

Previous systematic literature reviews examining pain management have explored racial and ethnic differences in a number of different healthcare contexts. However, none have focused specifically on advanced disease or palliative care, where the prevalence of pain associated with some disease can exceed 90% [36]. Most studies have been solely based within the USA: Perry et al. found higher pain scores in pre- and post-operative pain for people from ethnically diverse backgrounds in the USA [37]. Anderson et al. found racial disparities in pain across American healthcare, including; acute, chronic, cancer, and palliative pain care [16]. Kwok et al. reviewed the international literature on cancer pain, observing variations in pain outcomes across ethnic groups [38]. Four literature reviews examined the large disparities by race in the USA, they all reported that Black and African American patients were less likely to receive pain medication, or have access to analgesics [39–42]; and more likely to have their pain underestimated by physicians [40]. However, Santos Salas et al. undertook a meta-analysis of non-pharmacological pain interventions in the USA, and found no statistically significant differences in pain intensity between people from ethnic minority groups and the White group [43]. Reviews investigating self-management of pain have revealed ethnic differences in coping strategies, behaviours, communication and delays in help-seeking [42, 44–46]. Previous systematic literature reviews examining race and ethnicity within palliative care have investigated issues such as, access to hospice and advanced care planning, but none have specifically focused on pain management in advanced disease [47–51].

### A cross-national approach to the literature

Questions about advanced disease, race and ethnicity are becoming increasingly important globally as national populations grow and change. Many countries are projected to undergo population ageing [52–58], resulting in associated increases in disease, multimorbidities, and a greater dependence on health and palliative care services [59]. Some countries are projected to increase in racial and ethnic diversity: For example, in the USA by 2060 the number of people from the non-Hispanic White population is predicted to decline, while the fastest growing groups comprise people who identify with more than one racial category [60]. In New Zealand by 2043, the ‘European or Other’ ethnic group is projected to be the only group to decrease its population share [61]. In Canada, it is estimated that ‘visible minorities’ will grow from 19.6% in 2011, to 39.9% in 2036 [62]; and in England and Wales, it is expected that the non-White population will rise to 29% of the total population by 2051 [63]. Conversely, in Asian countries such as Singapore and Hong Kong, the proportions of ethnic groups among the resident populations are relatively stable, although both countries are expected to undergo population ageing [64, 65]. Trends in global migration can also impact upon health and palliative care services. Migration reports show a growing number of older people from diverse backgrounds in many countries internationally [66, 67]. This mixed methods systematic literature review therefore takes a cross-national approach to address questions of pain management, advanced disease and race/ethnicity.

### Developing a cross-national approach

Systematic literature reviews often search international literature without taking an explicitly cross-national approach, for reviews focused on medical and scientific concepts with consistent definitions, this method is generally unproblematic. However, questions of race and ethnicity are more complex internationally. Racial and ethnic groups names can vary from country to country, and even within countries are heterogenous [68]. The terms ‘race’ and ‘ethnicity’ have no agreed upon definitions within the English language, and are often used interchangeably. Most sociologists understand both race and ethnicity to be socially constructed terms: race is generally used to refer to the more physical aspects of heritage such as skin colour; while ethnicity tends to refer to cultural aspects such as language, religion and traditions [69, 70]. A sociolinguistic approach to race and ethnicity sees language, not just as a mirror to describing identity, but as part of the process of racial or ethnic identity formation [71]. Thus focusing on a shared language allows for some international comparison, not as like-for-like, but with some semantic overlap to allow for contrasts and comparisons across different geographical places. The aim of a cross-national approach is to allow for international comparisons and contrasts whilst recognising national context, and to compare countries where different ethnic groups are minoritised [72]. In particular, we aimed to add in insights from outside Europe and North America. We acknowledge this remains inadequate for a full understanding of racial and ethnic identity groups, particularly for those who are multilingual [73], it is a limited expansion to a single country English-language only review.

A note on terminology: In the main body of this paper, racial and ethnic groups are reported according to the American Psychological Association (APA) style guidelines [74]. For the mixed methods findings, the original racial and ethnic group names used by the article authors have been retained in the quotations. This is to take account of the time and context of the research undertaken. For the results of the primary outcome measure, a table of summary racial and ethnic groups has been created for comparative analysis across categories.

### Rationale and objectives

Pain is a common and distressing symptom prevalent in many advanced diseases and conditions. Racial and ethnic inequalities in pain management have been long noted, but little research has focused on advanced disease and the palliative care context is not well described. This issue is becoming increasing important internationally, as the populations of many countries are undergoing diversification and ageing. We therefore undertook a mixed methods cross-national review of the literature. To investigate differences in receiving pain medication for people with advanced disease, we reviewed studies examining statistical or clinical differences in measures of pain medication between people from different racial and ethnic groups. To our knowledge this is the first systematic literature review to examine the international literature on pain management by race and ethnicity for people with advanced disease across all disease groups.

Main objectives:

- To use quantitative evidence to determine whether there are statistically or clinically significant differences in receiving pain medication by racial and/or ethnic group for people with advanced diseases.
- To undertake a mixed methods synthesis and Thematic Analysis [75] of the cross-national qualitative and quantitative evidence together.
- To use the mixed methods synthesis to explore the context of the quantitative findings, and to explore more broadly the issues and themes in pain management for people with advanced diseases from different racial and ethnic groups.

## Methods

### Protocol and registration

The protocol was prospectively registered with PROSPERO (CRD42020167890) [76]. In this paper we describe the findings concerning pain medication and pain management issues. In a separate publication we will report on the results pertaining to differing pain levels and experiences of pain.

### Study design

A mixed methods systematic review: including quantitative tabling of the primary outcome measure; and a mixed methods Thematic Analysis [75] of qualitative and quantitative data together, utilising the conceptual/thematic matrix from Kavanagh et al [77]. The international literature was searched using a cross-national approach, and reporting follows PRISMA 2020 guidelines [78].

### Philosophical approach

The philosophical approach underpinning this mixed methods review is represented by pragmatism [79, 80]. A pragmatist approach to research is based on the proposition that the philosophical and methodological approaches should serve the question being investigated. ‘Pragmatist researchers’ are problem-oriented, they treat the research question as more important than the methods they use, and the paradigms underneath them [81]. Maxcy suggests that pragmatism is both a method of inquiry, and a device for settling the battles between research purists and more practical-minded scientists [82]. In this review we take a pragmatist approach to the evidence, and synthesise both the quantitative and qualitative methods to address the aims and objectives. There is dual value in both the qualitative and quantitative evidence. The quantitative research measures the relationships between race, ethnicity and pain medication. Whilst the qualitative research provides context, and explores in detail participants’ own experiences and perceptions.

### Study setting: selected countries

The criteria for countries included in the cross-national comparison are: (1) Top 20 of ‘very high’ United Nations (UN) Human Development Index (HDI) – to allow for comparisons of similar population challenges in the coming decades, and differing health and political systems. (2) English spoken as a major national language.

- Australia
- Canada
- Hong Kong
- Ireland
- New Zealand
- Singapore
- United Kingdom
- United States

### Eligibility criteria

The eligibility criteria are included in Table 1 below.

**Table 1:** Cross-tabulation illustrating eligibility criteria for included studies

	**Inclusion**	**Exclusion**
Setting	•Top 20 UN Human Development Index (HDI) ranked ‘Very High’ •English as one national language •Any healthcare setting or service, e.g. primary, emergency, palliative, community etc	•Country outside of UN HDI ‘Very High’ ranking •Non-English language speaking
Population	•Patients, or proxy discussions of real patients •Advanced stage of any incurable lifespan-limiting condition or disease •All ethnic, racial or cultural groupings as defined within the paper •With pain related to disease or treatment •Adults 18 years +	•Discussions of hypothetical patients •Non lifespan-limiting conditions, or curable disease •Acute injury or trauma patients only •Chronic, psychological or spiritual pain only •Children/paediatric
Study design	•Any qualitative or quantitative method •Vignette or preference studies that are based on real life experience of pain and disease/condition	•Vignette, hypothetical or preferences of a healthy population •Case series and single case reports •No new empirical data or new analyses
Comparison	•Either: A comparison between two or more racial or ethnic groups: •OR if focusing on a single group: An explicit focus and description of racial or cultural or ethnic or linguistic factors as they pertain to pain management within that group	•A paper with one racial or ethnic group which contains no explicit focus, and no description of racial or cultural or ethnic or linguistic factors as they pertain to pain management within that group
Publication	•Publication from year 2000 onwards •Published within a peer-reviewed journal •English language publication	•Data older than 10 years before first eligible publication date of the year 2000 •Abstract publication only •Unpublished •Non-English language •Opinion pieces, reviews, editorials
Quantitative primary outcome measure	•Statistically or clinically significant difference in the level of received pain medication by racial or ethnic group •Including both standardised measures such as Pain Management Index (PMI); and within-study designed measurements	•Qualitative •No comparison between groups

### Inclusions and exclusions

Whilst pain is a biopsychosocial process [13], studies which focus on pain experienced by people as solely psychological, or solely spiritual, have been excluded. This is because the mechanisms for managing these important aspects of pain are different, and outside the scope of this review. Any location of healthcare setting or service has been included if the patients have advanced disease. This is because some literature indicates that those from ethnically diverse groups do not have equal access to specialist palliative care services [83–85], and may have their disease manged elsewhere. Pain management for people under 18 years old was excluded because paediatric pain management services are often separate to adult services, and also outside the scope of this review. Studies since the year 2000 have been included to provide a sufficiently broad yet focused time frame, as the racial and ethnic groups of countries change over time.

### Information sources and search strategy

Searches were undertaken of five key electronic databases (Medline [2000-Present], AMED [2000-Present], CINAHL [2000-Present], EMBASE [2000-Present], PsychINFO [2000-Present]). The key databases were last accessed on 24/08/21. (Full search strategies are included in Supplementary Table 3).

### Supplementary approaches to identify literature

Additional published literature was sought by handsearching key journals, websites and the ETHoS thesis database. This was last undertaken in September 2021. Grey literature was then used to locate further published evidence by the authors, and for hand searching of the indexes. Where eligible abstracts only were located, authors were emailed to request full publication details. Key experts were identified and emailed to ask for recommendations on relevant papers.

### Selection and data collection

At least two members of the research team independently screened titles, abstracts, full papers and extracted the data (GC, EC and JC). A pre-designed data collection form was used by reviewers to extract data on both the primary outcome measure, and for the mixed methods analysis. Conflicts were resolved by discussion, or discussion with the full review team (GC, EC, JC, JK, SA, MB).

### Data items

The primary outcome measure was: Differences in receiving pain medication as part of standard care (not an RCT or trial) by ethnic or racial group for people with advanced disease. The included measurement tools were: validated tools for standardising pain management scores, e.g. PMI (Pain Management Index); and in-study designed methods to compare proportions of patients. Any racial or ethnic group comparison as defined within the paper was included. Studies with missing data on the primary outcome were excluded from the primary outcome analysis. The level of statistical or clinical significance is as reported within the paper, and recorded in the cross-tabulation where available. Both cross-sectional and longitudinal studies were included, if the patients already had advanced disease at the first time point. For longitudinal studies, each reported follow-up time point measure was included in the cross-tabulation to compare differences at any time point. The effect measures were: Statistically or clinically significant differences by ethnic or racial group.

Data included in the mixed methods analysis comprised: Qualitative or quantitative data comparing pain management (self or clinical) in two or more ethnic or racial groups; or focusing on a single ethnic group if there was an analysis or discussion of racial, cultural, ethnic, or linguistic factors as they pertain to pain management within that specific group.

### Risk of bias and weight of evidence

Studies were evaluated for risk of bias using the Critical Appraisal Tools checklists from the Joanna Brigg’s Institute (JBI) [86]. The domains assessed for qualitative and quantitative studies varied, including a focus on study populations, outcome measures, and confounding factors for quantitative studies and congruity of methodology for qualitative studies [86]. Rating was undertaken independently by at least two team members (GC, EC, JC). Conflicts were resolved by discussion. Studies scoring less than 70% on the relevant checklists were excluded. Those that remained were categorised as ‘High’, ‘Medium’, or ‘Lower’ quality. Gough’s Weight of Evidence (WoE) includes the domains of risk of bias, as well as the relevance and focus of the data to look at certainty of the evidence overall [87]. The JBI risk of bias assessments were integrated into the WoE framework. The further WoE categories were independently rated by at least two reviewers (GC, EC, JC) and the total WoE score was used to rank the weight of evidence for each study. The WoE framework is reported for each study and used to undertake a sensitivity analysis.

### Data analysis and synthesis

The guidelines for mixed method data synthesis from Kavanagh et al. (2012) [77] were adapted to combine the qualitative and quantitative findings. This method of synthesis involves three stages:

- Stage 1 - A traditional systematic review of quantitative findings for the primary outcome measure. Quantitative results on the primary outcome were tabulated for synthesis. Meta-analysis was not undertaken because the measures and racial/ethnic groups were heterogenous. Data conversions: For the analysis of the primary outcome measure, summary inter-related racial and ethnic groups were tabulated together for analysis. No numerical conversions were undertaken. WoE was used for a sensitivity analysis by comparing the whole dataset with those rated ‘High’.
- Stage 2 - Thematic coding of all qualitative and quantitative studies. Two members of the research team (GC, EC) independently extracted and coded data using NVivo Plus™ software. Braun and Clarke’s thematic analysis [75] method was used to form the key themes and sub-themes.
- Stage 3 - Cross-study mixed-methods synthesis drawing together *Stage 1* and *Stage 2* to create a conceptual/thematic matrix of key themes. The key themes from the mixed methods analysis are reported. WoE was used for a sensitivity analysis by comparing the whole mixed methods dataset with those rated ‘High’.

### Public and Patient Involvement

Public and Patient Involvement (PPI) was sought on the findings and themes from this review. A seminar was held to discuss initial themes and findings from the review with four South Asian community patient representatives in Bradford, UK in February 2020. Four later individual telephone interviews were undertaken with patient representatives from Black, African and/or Caribbean groups. These were carried out from Summer to Autumn 2020. PPI representatives’ perspectives, comments and critique have informed the analysis and are included in the discussion section.

## Results

### Included papers

**Fig. 1:**
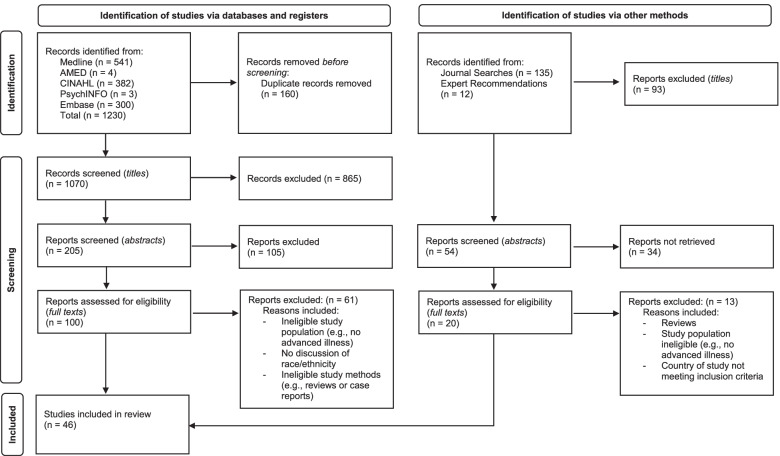
PRISMA 2020 flow diagram of included papers

Electronic searches yielded 1230 titles, 160 were excluded as duplicates. Screening was undertaken of 1070 titles, 205 abstracts and 100 full texts, leaving 39 full papers from the electronic searches. Expert recommendations, grey literature, hand searching of key journals, and the index searching of previous systematic reviews yielded seven further full papers for inclusion. In total 46 full papers were included for the primary outcome analysis and the mixed methods findings. The search process is summarised in Fig. 1 below.

Of all 46 included papers, 41 were based in the USA (89%) [85, 88–127], three in the UK (7%) [128–130], one in Australia (2%) [131], and one in New Zealand (2%) [132]. No papers were identified from Canada, Ireland, Hong Kong or Singapore. Ten studies were rated ‘High’ Weight of Evidence (WoE) (22%) [88–94, 96, 128, 131]. The characteristics of the included studies are summarised below in Table 2. Some studies which focused on pain management and a single ethnic group were excluded, as they did not explicitly examine the relationship between ethnicity, race, culture or language as it pertains to pain management (e.g. Yeager et al.) [133].

**Table 2:** Crosstabulation illustrating summary of all included studies (*N* = 46)

No	First author Country Year	Study population and (N)	Ethnic groups^1^ (%)^2^	Disease groups	Setting	Summary method and aims	WoE^3^
1	Anderson USA 2000 [88]	Patients (108) Clinicians (55)	Patients: African American (41%) Hispanic (59%) Clinicians: White (68%) African American (17%) Asian/ Pacific Islander (11%) Other (4%)	Cancer	Hospital—outpatients	Patient questionnaire survey to determine pain needs, including pain measures and PROMs. Clinician survey on pain management to determine attitudes	H
2	Anderson USA 2002 [89]	Patients (31)	African American (45%) Hispanic (55%)	Cancer	Hospital – outpatient oncology clinic	Qualitative structured interview with pain measure rating to explore barriers to pain management	H
3	Bell USA 2011 [90]	Patients (4,658)	White (43%) Asian (34%) Hawaiian/Pacific Islander (18%) Other (5%)	Cancer	Hospital—inpatient	Clinical data analysis including pain measures, to examine ethnic disparities in pain outcomes after palliative care consultations	H
4	Booker USA 2020 [91]	Patients (64)	African American (50%) Caucasian (50%)	Cancer	Hospice—inpatient	Secondary matched data analysis examining ethnic differences. Data drawn from an RCT of cancer pain management interventions	H
5	Green USA 2009 [92]	Patients (96)	White (71%) Black (21%) Native American (4%) Hispanic (3%) Arabic (2%) Black/Native American (1%)	Cancer	Hospital—outpatient	Longitudinal cohort pain survey to examine ethnic differences. Including PROMs and pain measures at 3 time points	H
6	Koffman UK 2008 [128]	Patients (45)	Black Caribbean (58%) White British (42%)	Cancer	Hospital inpatient, outpatient, community palliative care	Semi-structured qualitative interview to examine ethnic meanings attributed to cancer pain	H
7	Laguna USA 2014 [93]	Patients (385)	White (46%) Black (25%) Latino (29%)	Cancer Heart Failure Heart Disease Dementia	Hospital – from inpatient to outpatient	Longitudinal study of pain consultation intervention to examine ethnic differences, with data collected at 4 time points	H
8	Lamba USA 2020 [94]	Patients (17,957)	White (75%) African American (11%) Hispanic (7%) Asian (7%)	Cancer—Brain metastases	All – Medicare data	Retrospective analysis of data to evaluate prevalence of medication utilisation, and assess potential racial disparities in prescriptions of such medications	H
9	McGrath Australia 2006 [131]	Patients (10) Carers (19) Aboriginal health workers (11) Healthcare professionals (30) Interpreter (2)	Aboriginal (100%)	Not reported	Community	Qualitative semi-structured interviews to explore and document issues associated with pain management for Aboriginal people	H
10	Wilkie USA 2017 [96]	Patient-Caregiver dyads (161)	Patients: Caucasian (49%) African Amer (34%) Asian Amer (< 1%) Other (13%) Multi-race (2%) Unknown (1%)	Cancer	Home hospice care	Cross-sectional study of pain management barriers, data from an RCT of home hospice	H
11	Anderson USA 2004 [97]	Patients (97)	African American (43%) Hispanic (57%)	Cancer	Hospital—outpatient	RCT of pain management education intervention, including PROMs and pain measures. Evaluation of the efficacy for minority patients	M
12	Carter USA 2006 [98]	Physicians (40)	African American (60%) Caucasian (40%)	All EOL care	Primary care, oncology, cardiology	Semi-structured interview examining physicians’ perceptions of patients’ preferences, comprising quantitative and qualitative components	M
13	Cea USA 2016 [99]	Patients (3,918)	Non-Hispanic White (84%) Non-Hispanic Black (11.1%) Hispanic (3%) Other race/ethnicity (2%)	Cancer and non-cancer	Hospice discharge of home health and hospice	Secondary analysis of national hospice survey data, examining pain assessments, management and outcomes	M
14	Check USA 2016 [100]	Patients (883)	White (85%) Black (15%)	Cancer	All settings – Medicare data	Retrospective analysis of Medicare data to examine ethnic disparities	M
15	Chung USA 2016 [101]	Patients (161)	African American (9%) Asian/Other (9%) Caucasian (48%) Hispanic/Latino (33%)	Cancer COPD Heart Failure Liver Failure Organ failure	Hospital – inpatient	Retrospective clinical data analysis, including pain measures, to examine ethnic disparities	M
16	Edrington USA 2009 [102]	Patients (50)	Chinese American (100%)	Cancer	Hospital—outpatient	PROMs questionnaire survey with pain measures examining barriers to pain management	M
17	Fisch USA 2012 [103]	Patients (3,123) (2,026 in analysis)	In analysis White (77%) Hispanic/Latino (9%) Black (12%) Asian (1%) Other minority (1%)	Cancer	Hospital—outpatient	Prospective study of pain, symptoms and prescribing, with pain measurements at two time points	M
18	Fischer USA 2007 [117]	Patients (217)	African American (13%) White (68%) Hispanic White (9%) Other (1%) Unknown (10%)	Cancer	Hospital—inpatient	Retrospective analysis of clinical data from veterans’ hospital to determine ethnic differences in EOL care	M
19	Gerlach USA 2021 [104]	Patients (554,022)	Non-Hispanic White (88%) Non-Hispanic Black (8%) Hispanic (2%)	Cancer Heart disease Dementia, Respiratory disease Stroke	Hospices	Prevalence study of class-specific psychotropic and opioid prescriptions	M
20	Gurney New Zealand 2021 [132]	Patients (20,081)	Māori (20%) Non- Māori (80%)	Lung cancer	All – New Zealand Cancer Registry data	Retrospective analysis of data to describe access (and timing of access) to pain medications	M
21	Hwang USA 2004 [105]	Patients (89)	Caucasian (57%) African American (43%)	Cancer	Hospital	Retrospective data analysis of palliative care resource use in prostate cancer patients	M
22	Khosla USA 2016 [106]	Clinicians and healthcare providers (57)	South Asian (39%) Not South Asian (61%)	All EOL care	Healthcare systems, community, hospice and private practice	Qualitative interviews and focus groups on South Asian patients’ preferences	M
23	Koffman UK 2003 [129]	Family members and friends of patients (69)	Patients: Black Caribbean (49%) UK-born White (51%)	Cancer	Home or hospital death of patients	Structured questionnaire interview on patients’ symptoms before death	M
24	Kwon USA 2013 [107]	Patients (196)	White (75%) Black (15%) Hispanic (7%) Other (3%)	Cancer	Hospital—outpatients	Secondary analysis of data from an observational cohort study to examine barriers to pain management, including PROMs and opioid adherence data	M
25	Meghani USA 2021 [108]	Patients (32)	White (50%) African American (50%)	Cancer	National Cancer Institute	Qualitative, semi-structured interviews to describe cancer patients’ concerns while undergoing cancer treatment, and determine if concerns differ between African Americans and Whites	M
26	Mosher USA 2010 [109]	Patients (87)	African American (17%) Spanish speaking Latina (31%) English speaking Latina (11%) Caucasian (31%)	Cancer	Hospital—outpatient	PROMs questionnaire survey to examine the relationship between self-efficacy for coping and pain management/distress	M
27	Nedjat-Haiem USA 2012 [100]	Patients (24)	Latina (100%)	Cancer with depression	Medical centre—outpatient	Qualitative interview on Latin American patients’ sociocultural beliefs and health care, part of a wider RCT intervention on cancer and depression	M
28	Pinheiro USA 2019 [111]	Patients (23,091)	USA born non-Hispanic (88%) USA born Hispanic (4%) Non-USA born Hispanic (6%) Foreign born Hispanic (2%)	Cancer	All – National cancer and Medicare data	Secondary data analysis of cancer registry and Medicare data examining ethnic differences in breast cancer patients’ medication use	M
29	Rabow USA 2005 [95]	Patients (90)	Asian/Pacific islander (12%) Black/African American (18%) Latino (10%) White/Caucasian (53%) Other (7%)	Cancer COPD Heart Failure	Hospital—outpatient	Cohort study within a controlled trial of a palliative care consultation, including exploration of ethnic differences	M
30	Reynolds USA 2008 [112]	Patients (1,133)	White (77%) Minority (23%)	Dementia Stroke/CVA Heart Failure Diabetes COPD Cancer	Nursing homes	Observational cohort study. Clinical chart review of nursing home residents’ data to identify differences in end of life care	M
31	Rhodes USA 2007 [113]	Proxies of decedents (98,911)	Non-Hispanic Black (4%) Non-Hispanic White (96%)	Cancer, Dementia Heart disease Lung disease Frailty/Old age Other	Hospices	Survey design to examine whether racial differences in perceived care exist	M
32	Zapka USA 2006 [114]	Patients and race-matched physician for interview (90)	Caucasian (43%) African American (57%)	Cancer Heart Failure	Hospital – outpatient and primary care	Cross-sectional structured interview study using race-matched physicians interviewing patients on the topic of communication	M
33	Burgio USA 2016 [115]	Patients (6,066)	White (65%) African American (35%)	Cancer Dementia Lung Disease Heart Disease Kidney Disease Liver Disease Stroke HIV	Hospital—inpatient	Secondary data analysis of education intervention trial. Data collected after death to examine processes of care at EOL and ethnic differences	L
34	Campbell USA 2013 [85]	Patients, family members or caregivers (743)	Survey respondents: White (85%) African American (12%) Other (3%)	Cancer and non-cancer	Hospice – inpatient and community	Questionnaire survey to measure satisfaction with hospice services by patient/family ethnicity	L
35	Dhingra USA 2011 [116]	Patients (170)	Chinese American (100%)	Cancer	Private community-based oncology centre—outpatients	Describing the epidemiology of pain in Chinese Americans using clinical data analysis and PROMs measures questionnaire survey	L
36	Halpern USA 2019 [118]	Patients (8,438)	Non-Hispanic white (53%) Non-Hispanic black (21%) Hispanic (13%) Other/unknown (13%)	Cancer	All – Medicaid data	Retrospective analysis of Medicaid data examining pain management, state policy and patient characteristics	L
37	Kalauokalani USA 2007 [119]	Patients (67)	White (78%) Latino (9%) Asians (6%) Black (3%) Other (4%)	Cancer	Hospital—outpatient	Secondary data analysis of RCT examining whether patient coaching can reduce ethnic equalities in pain	L
38	Kypriotakis USA 2014 [120]	Patients (196)	Black (33%) White (66%)	Cancer	Hospital—outpatient	Secondary data analysis examining ethnicity and preferences for care. Data drawn from RCT of coping and cancer intervention	L
39	Monroe USA 2010 [127]	Patients (55)	African American (29%) Caucasian (71%)	Dementia with cancer	Nursing home resident	Retrospective data review to examine ethnic differences in pain management at the end of life.	
40	Patel USA 2020 [121]	Patients (142) (43 for sub study)	White (63%) Black (35%)	Cancer	Clinic—outpatient	Quasi-experimental study assessing the impact of clinical pharmacy assessments with and without pharmacogenomics	L
41	Rhodes USA 2012 [122]	Bereaved family members (11,892)	Deceased patients: African American (100%)	Cancer Dementia Heart Disease Lung Disease Frailty/ Old Other	Hospice	Secondary analysis of national survey data comparing family members’ perceptions of care with the proportion of African-Americans at hospice	L
42	Rolnick USA 2007 [123]	Patients (421)	White (83%) African American (8%) Asian (4%) Hispanic (4%) Native American (< 1%)	Ovarian cancer	Hospital	Retrospective analysis to report on medication use for pain management during the last six months of life	L
43	Sadler UK 2009 [130]	Patients (244) In analysis: (231)	Caucasian (70%) Black (14%) Asians (10%) Not included in analysis: Chinese (1%) Unknown (4%)	Cancer	Hospital	Retrospective clinical audit examining influence of ethnicity on presentation, symptomatology and outcomes in oesophageal cancer	L
44	Saphire USA 2020 [124]	Patients (16,246)	White (81%) Black (8%) Hispanic (5%) Asian (6%)	Lung cancer	Hospital—outpatients	Retrospective cohort study using data to examine patterns of symptom management medication receipt at EOL	L
45	Strassels USA 2006 [125]	Patients (347,555) (156,887 included in pain analysis)	Asian or Pacific Islander (1%) African American (5%) Hispanic or Latino (2%) Native Amer (< 1%) Caucasian (54%) Other (< 1%) Missing (38%)	Cancer Heart Disease COPD Unspecified	Hospice	Retrospective analysis of national clinical and administrative data to describe demographics, clinical characteristics, and pain intensity of persons who received hospice care	L
46	Wieder USA 2014 [126]	Patients (360)	African American (48%) Hispanic (33%) Caucasian (13%) Not included in analysis Asian (5%) Not specified (2%)	Cancer	Hospital – inpatient, outpatient and emergency depart	Clinical data review to examine prescription coverage and the prescribing of long-acting opiates to minority patients with cancer pain	L

^1^Ethnic and racial group names as stated in original paper

^2^Rounded to 0 decimal places

^3^*WoE* Gough’s Weigh of Evidence Framework. Category D – total score. *H* Higher, *M* Medium, *L* Lower

For the primary outcome analysis only: Eighteen papers were located that examined differences in receiving pain medication as part of standard care by ethnic or racial group [88, 89, 94, 99, 100, 103–105, 109, 111, 112, 117, 118, 123, 124, 126, 127, 132, 134]. Seventeen of these papers were based in the USA (94%) [88, 89, 94, 99, 100, 103–105, 109, 111, 112, 117, 118, 123, 124, 126, 127], and one in New Zealand (6%) [132]. Racial and ethnic groups included in the papers have been tabled together below into analysis groups (Table 3 below).

**Table 3:** Table showing racial and ethnic groups summary analysis groups from included papers in primary outcome measure analysis (*N* = 18)

Original racial and ethnic groups names in primary outcome papers	Countries	Summary analysis group name	No. of studies
White Caucasian White and non-Hispanic Non-Hispanic White	USA	White	14
Black African American Non-Hispanic Black	USA	Black and African American	13
Hispanic Spanish speaking Latina English speaking Latina Hispanic White US-born Hispanic Foreign-born Hispanic	USA	Hispanic	10
Minority Other All other race Not specified	USA	Minority and other	5
Asian Asian, Other	USA	Asian and other	3
USA born non-Hispanic Foreign born non-Hispanic	USA	Non-Hispanic	1
Māori	New Zealand	Māori	1
Non-Māori	New Zealand	Non-Māori	1

### Primary outcome measure: differences in receiving pain medication

The primary outcome measure we examined was differences in receiving pain medication as part of standard care by ethnic or racial group for people with advanced disease. Six studies reported significant differences in receiving pain medication between people from different racial or ethnic groups (6/18, 33%) [94, 99, 103, 104, 111, 124], and two studies had mixed findings (2/18, 11%) [118, 126]. Of these: 5/8 (63%) reported Hispanic patients were significantly less likely to receive pain medication as compared to White patients, or all other groups [99, 111, 118, 124, 126]. In 3/8 (38%) studies, Black and African American patients were significantly less likely to receive pain medication compared to the patients in the White group [104, 118, 124]; and in 3/8 (38%) studies, Asian patients were significantly less likely to receive medication compared to White patients, or other groups [94, 124, 126]. No studies reported White patients were significantly less likely to receive pain medication. (Cross-tabulation of findings in Supplementary Table 1).

Ten studies found no statistically significant differences between people from different racial or ethnic groups (10/18, 56%) [88, 89, 100, 105, 109, 112, 117, 123, 127, 132]; and the New Zealand based study did not find a significant difference between patients from Māori and non-Māori ethnic groups [132].

Only 4/18 (22%) papers accounted for the level of pain reported by the patient when calculating medication differences [88, 89, 103, 109]; 3/4 (75%) of these reported no significant difference [88, 89, 109]. A further five papers considered whether the patients had reported any pain, but not the level of pain experienced [99, 112, 117, 123, 126]; 3/5 (60%) of these reported no significant difference [112, 117, 123].

There were no trends by study setting. Seven studies were set in oncology or non-palliative settings [88, 89, 103, 109, 117, 124, 126], 3/7 found significant differences or mixed results in pain medication by racial or ethnic group [103, 124, 126]. Five studies were based in palliative care or nursing home settings [99, 104, 105, 112, 127], 2/5 reported significant differences in pain medication by racial or ethnic group [99, 104]. Six were set across all healthcare settings, or included data from all settings [94, 100, 111, 118, 123, 132], 3/6 found mixed or significant differences in pain medication by racial or ethnic group [94, 111, 118].

#### Certainty of evidence

Three studies were rated ‘High’ weight of evidence (17%) [88, 89, 94]; two found no significant differences in pain medication between people from different racial and ethnic groups [88, 89], and one observed significant differences [94]. As 17/18 (94%) studies were based in the USA, a cross-national comparison was not possible. Overall there is not enough high-quality evidence to draw a conclusion on the primary outcome measure.

### Mixed methods synthesis

Forty-six papers were included in the mixed method analysis (Table 2 above). A conceptual/thematic matrix was created (Supplementary Table 2). There were three key themes: Patient and family perspectives; Barriers to pain management; Service level and structural issues.

### Patient and family perspectives

#### Fears and concerns

The literature showed that many patients were challenged by fears and anxieties about pain medication. Side effects were a concern for many people from diverse ethnicities across the USA, UK and Australia [88, 89, 96, 102, 106, 108, 128, 129, 131]. Common complaints included constipation, confusion, sleepiness and drowsiness [106, 108, 128, 131]. For South Asian patients in the USA, some healthcare providers felt that fears about the effects of pain medications could be more acute within South Asian communities, as they could interfere with family leadership:

“*There is a pretty significant fear of not wanting to lose your mind from pain meds. And I think that’s a fear across the board, but more so in peoples from* [South Asia], *where elderly people are looked upon as the matriarch or the patriarch of the whole family and do want to maintain their mental faculties as much as possible, even at the expense of having some pain.”* (USA South Asian physician) [106].

Similarly, in a study of indigenous Australian patients, McGrath reported that families worried pain medication could interfere with end of life traditions between older and younger generations: [131].

“*…in Aboriginal culture the passing on of knowledge at that stage of life is a key component to the cultural survival. So there was a key concern that people would be leaving without passing on the knowledge*.” (Healthcare worker, Australia) [131].

Six studies reported that fears of addiction and tolerance were a significant concern for many people from ethnically diverse communities in the USA and Australia [89, 96, 102, 106, 108, 131]. Edrington et al. observed that concerns about tolerance were the number one barrier for Chinese American patients, but only the seventh highest concern for White American patients [102]. Khosla et al. reported some doctors believed that fear of addiction was a deeply rooted cultural issue for South Asians, because of the problems with addiction in parts of South Asia:

“*I know in Pakistan, heroin addiction is terrible ... Opiates ... are so easily available on the free market*.” (USA South Asian Physician) [106].

However, other clinicians in the same study felt that fear of addiction was an issue for patients across all ethnic groups, it was just nuanced by patients’ own cultural context [106]. In a qualitative study of African American and White cancer patients; fears about tolerance and addiction were a significant concern for both groups: [99].

“*All these pills, if you look on that—get on that system and see all the stuff I’ve been on, you would say it’s a wonder this woman is not a junky*.” (African American patient with cancer) [99].

Similarly, another USA study found no significant difference between Black and White patients’ preferences for pain relief at the end of life. [112] Four studies found that some patients were not adhering to their schedule, or stopping their medication altogether [88, 89, 108, 128].

#### Unmet pain management needs

Five studies reported that some patients wanted stronger medication, or had their pain underestimated by physicians [88, 89, 97, 128, 131]. The evidence showed that family members were also concerned about unmet needs. A UK study found that, compared to White British families, significantly fewer bereaved relatives of Black Caribbean patients felt their healthcare provider had tried hard enough to relieve their relative’s pain [129]. A USA survey reported that the relatives of African American decedents were more likely to have concerns about unmet pain needs compared to non-Hispanic White relatives [113].

#### Self-determination

Many patients across the included countries felt a sense of self-determination towards their pain management, and believed they should not be reliant on medication alone to cope. This included African American, South Asian and Hispanic patients in the US, Black Caribbean and White British patients in the UK, and Indigenous patients in Australia [88, 89, 96, 106, 108, 131]. Anderson et al. observed that over 90% of African American participants agreed with the statement they should ‘be strong’ and not lean on pain medication [89]. A UK study reported that patients from both Black Caribbean and White British groups viewed pain as a challenge, a test, or an enemy to be overcome, fewer patients felt they could not meet these challenges [128]. One Black Caribbean patient who viewed their pain as a challenge, felt they could personally negotiate their emotions and put their own pain into a manageable context:

“*It makes a difference to* (me) [going for respite care]…*it helps me to realise that there’s other people is worse than me and is suffering and is worse than me, so that brings me back to reality*.” (UK Black Caribbean patient with cancer) [128].

### Barriers to pain management

#### Information and misconceptions

Patient information and misconceptions about pain were investigated in five papers [88, 96, 110, 119, 131]. A USA study observed that patients from non-White groups had significantly higher pain misconception scores [119]; and another study showed that 43% of African American patients and 55% of Hispanic patients wanted more information about pain medication [88]. A qualitative study of Hispanic cancer patients observed that some underestimated the seriousness of their disease as a consequence of a lack of pain:

*“No, I didn’t feel anything, nothing. I didn’t get itchiness, no burning. That’s why it kept growing, because I didn’t, well, it didn’t hurt. But, why didn’t it hurt? The lump just grew, but it didn’t hurt.”* (USA Hispanic patient with melanoma) [110].

#### Doctor-patient communication

Some communication difficulties were related to language barriers, this was reported for Chinese American and Spanish speaking cancer patients in the USA [102, 109, 110, 116]. In one study of Spanish speaking Latina patients, many felt they were not getting all the information they needed about their condition, despite interpreters being available [110]. When family members acted as interpreters, some patients worried the information being given to them by family members was being filtered:

*“…my daughter is the one that speaks English, she’s the one that speaks with the doctor. And uh, but I feel sometimes that she doesn’t tell me everything*.” (USA Latina patient with breast cancer) [110].

Communication issues were not just related to language; cultural and social issues were also important. Two USA studies of Hispanic and African American cancer patients revealed that people felt they should wait until their pain was severe (level 7–10 on a 0–10 numerical scale), or their symptoms became serious, before reporting their pain to their healthcare provider [89, 110]. Although one study found no significant racial differences in pain communication scores [107]. Cultural stoicism was also reported as a communication barrier for USA South Asian [106] and Indigenous Australian [131] patients. In a study of African American and White cancer patients in the USA, both groups felt communication with healthcare providers could be too impersonal, and desired more individualised options to manage their pain: [108].

“*…this is what makes it impersonal. You’re not speaking about me. You’re speaking about a guideline that you have made up. How is that supposed to comfort me?... It’s not always about a statistic. It’s about the individual*.” (USA African American patient with breast cancer) [108].

#### Racial and ethnic stereotyping

Two studies discussed stereotyping. In a study of USA healthcare providers’ views, participants noted the perception of USA South Asian patients as reluctant to use pain medication, however they warned against this stereotyped view [106]. In a study of African American and Caucasian doctors’ perspectives, some doctors from both groups appeared to hold stereotyped views about Black and African American patients:

“*Blacks seem to be stronger with coping than Whites, maybe because of life experience..*.[Black patients] *can cope because of struggle… seems harder for Whites to cope”* (USA African American physician) [98].

*“Some slight differences..* [Black and African American patients] *will tolerate pain longer before coming to the doctor”* (USA Caucasian physician) [98]

Although other physicians in the same study pointed to other factors they felt were more important for pain management, such as age and financial issues [98].

### Service level and structural issues

#### Differences in the utilisation of standard pain management care

Two studies found that Black and African American patients were significantly less likely to receive an initial assessment for pain on admission, compared to White and Caucasian patients [91, 99]; and one reported that only 25% of African American patients and 29% of Hispanic patients indicated their doctor or nurse had used a pain scale for assessment [89]. One study reported on issues of the timing of pain management. A New Zealand study of opioids, showed that Māori patients were significantly more likely to get access pain medications late, only in the last two weeks before death [132].

#### Pain outcomes following treatment

Four USA-based studies reported African American, Hispanic or non-White patients had significantly less improvement in pain after standard pain management care [92, 93, 99, 101]; and one reported Hawaiian and Pacific Islander patients had greater improvements [90]. Within research studies: Four USA studies trialled palliative care interventions: Two studies did not report significant improvements in pain by racial group [97]; one patient education intervention observed significant improvements for ethnically diverse patients [119]; and a pharmacy intervention resulted in significantly less pain improvement for non-White and Black patients [121].

#### Research based studies

Three longitudinal studies examined differences in medication use as part of multifaceted palliative care research interventions: One showed no significant differences for people of different racial groups after the intervention [95]; another found no differences in pain medications given to people by racial or ethnic group [91], and another found African American patients were significantly less likely to receive opioid medications compared to White patients [115].

#### Racial/ethnic interactions and dynamics within healthcare

Two USA studies explicitly investigated racial dynamics and interactions within healthcare settings: Zapka et al. compared the race of the physician with the race of the patient [114]. The study showed that African American patients were significantly less likely to receive pain management under the care of an African American physician, while ‘Caucasian’ patients were significantly more likely to receive treatment for their pain, whether under the care of a ‘Caucasian’ or African American physician [114]. Rhodes et al. observed that family members of African American decedents were less likely to have concerns about unmet pain needs, when their relative was in a hospice with a high proportion of African American patients [122].

### Certainty and sensitivity of evidence

Of all 46 included studies, ten studies were rated ‘High’ WoE (22%) [88–94, 96, 128, 131]. Examining only the studies rated ‘High’ did not affect the three key themes. However, it did remove the subthemes on racial and ethnic stereotyping, and racial/ethnic dynamics and interactions. It also substantially reduced the evidence base on patient and family perspectives, fears and concerns.

## Discussion

### Evidence in the primary outcome measure analysis

In the analysis of the primary outcome measure, only three studies were rated as ‘High’ weight of evidence (17%) [80, 81, 86], and overall there was not enough high-quality evidence to draw a conclusion. Issues with the quality of the evidence included, only four of the eighteen studies (22%), incorporated the patients’ own reported levels of pain into the medication difference calculations [88, 89, 103, 109]. Additionally, two papers that reported no significant differences in the pain medication received, described elsewhere in the paper that they had found significant differences in the levels of pain experienced by patients from ethnically diverse backgrounds [109, 127]. This potentially indicates there should have been a respective difference in the medication prescribed, or that the medication was not working in the same way for these patients. The mixed methods analysis highlighted the importance of intersectional factors, such as gender and socioeconomic status, in understanding the relationship between race, ethnicity and pain management. However, these factors were not included in the primary outcome analysis and may account for the mixed results.

### Differences between racial and ethnic groups in different countries

There were not enough papers from non-USA countries identified for either the primary outcome measure (1/18, 6%) [132], or the mixed methods synthesis (5/46, 11%) [128–132], to undertake a cross-national comparison between countries. This means that comparisons between countries, and the minoritisation of different ethnic groups across countries, were not possible. However, comparisons within the USA between different racial and ethnic groups were possible. For the primary outcome measure: Of the eight papers observing some significant differences: 5/8 (63%) reported Hispanic patients were significantly less likely to receive pain medication compared to all other groups, or to people from the White group [99, 111, 118, 124, 126]. The Hispanic and Latin experience in palliative care is still an under researched area and requires greater attention. No studies reported White patients were significantly undertreated for pain. For the mixed methods synthesis: Those from ethnically diverse backgrounds in the USA may not receive the same level of pain relief from their treatment [83, 84, 90, 92]. Black and Hispanic people in the USA may be less likely to receive adequate pain assessment [89, 91, 99]. Patients from all racial and ethnic groups had fears about side effects [88, 89, 96, 102, 106, 108, 128, 129, 131], but people from ethnically diverse groups in the USA and Australia were more likely to express fears of addiction or tolerance [89, 96, 102, 106, 108, 131].

### Pain communication and bias

Two USA studies showed that significantly fewer Black and African American patients received an initial pain assessment [91, 99], and a further paper found that a pain scale had only been used for 25% of African American patients and 29% of Hispanic patients [89]. The lack of standardised measurement, potentially means that Hispanic, Black and African American patients’ pain is not being recognised initially, and appropriate referrals to palliative care are not being made. Even when pain was explicitly reported by people from ethnically diverse groups, it was not always recognised the same way as pain reported by people from White groups. In a UK study of oesophageal and gastric cancer, the authors observed that Black and Asian patients were more likely to present with abdominal pain compared to White British patients however this was less likely to trigger an urgent referral [130].

Pain is a subjective experience, and the lack objective criteria means it is open to medical ambiguity and sociopsychological influences [14]. The lack of standardised tools may allow for the influence of implicit bias and stereotypes. Two studies in the mixed methods synthesis found evidence that some doctors hold some stereotyped false beliefs; about pain tolerance for Black and African American people [98], and regarding USA South Asian people’s willingness to use pain medication [106]. The wider literature outside of palliative care has also shown that some clinicians hold false beliefs about biological differences in pain experience between Black people and White people (such as “Black peoples’ nerve endings are less sensitive than white peoples’ nerve endings”), and these beliefs have been shown to influence treatment decisions [35, 135]. A 2019 USA study of lay people asked participants to identify pain expressed in photos, and found that White participants more readily recognised pain on White people’s faces compared to Black people’s faces [136]. Due to the ambiguities around pain, communication between doctor and patient is crucial. However, pain communication emerged from the synthesis as another challenging area. Four USA-based studies showed that patients’ pain was underestimated and many patients wanted more or stronger medication [88, 89, 97, 128, 131]. Moreover, even when there were no ethnic differences in pain medication, there was still a substantial amount of undertreatment for pain. For example, in two studies of African American and Hispanic patients, undertreatment ranged between 28–36% of all patients [88, 89]. Communication issues highlighted by the evidence were; language barriers [102, 109, 110, 116], and social/cultural communication issues such as ‘cultural stoicism’ and only discussing severe pain [89, 106, 110, 131].

There is a need to recognise greater variations in pain expression, different ways of communicating about pain, and to build trust so people feel comfortable communicating about pain. Efforts to decolonise medical education and curricula are attempting to do this [14, 137]. Decolonising pain management is not just about the recognition of different expressions of pain, though this is important. It is also an anti-racist stance against stereotyping, a decentring of the European, White male standard of pain expression and an aim to re-centre the perspectives of groups who have been historically minoritised [14, 72, 137]. Palliative care has traditionally not engaged with issues around ethnicity, race and racism. According to Gunaratnam, this may be due to the historic and continued development of palliative care by charismatic largely White leaders [138, 139]. This review only found two USA papers engaging with racial and ethnic stereotypes [98, 106], and two USA paper explicitly examining the racial and ethnic dynamics of palliative care services [114, 122] The wider literature from the UK has highlighted the lack of research in this area and the poorer palliative care provision for those from Black, Asian and ethnically diverse groups [83, 140–142]. However, changes to clinical practice and research have been slow, and few of the reports’ recommendations have been implemented [30–33]. A recent editorial in *Palliative Medicine* has addressed racism within UK palliative care, calling for; a recognition of racial and minority ethnic disadvantage, an understanding of racism, better data on ethnicity, and anti-racist action through partnership [138]. Similarly, a recent American article asks pain physicians to do more to tackle racism in pain management, the authors recommend strategies to reduce implicit bias and create a culture of safety within palliative care [143].

### Culturally competent communication

The mixed methods findings highlight challenging issues for discussion between doctor and patient, such as fears about side effects [88, 89, 96, 102, 106, 108, 128, 129, 131] and concerns about addiction or tolerance [89, 96, 102, 106, 108, 131]. Although these discussion may be already taking place for many patients, the evidence that some patients are deliberately stopping or reducing their medication covertly [88, 89, 108, 128], indicates that their fears are not being addressed, or communicated to their healthcare provider. For patients with slower progressing conditions, or those earlier in the disease trajectory, fears about addiction may be well-founded. The USA has been facing “an opioid crisis” [144]; USA deaths from prescription opioids have tripled between 1999 and 2007, and are also increasing in many other countries, including the UK, Canada and Australia [145]. However, for most patients with advanced disease, this may reflect a denial or a lack of knowledge about their prognosis and a lack of understanding about the aims of palliative treatment. It is also representative of the difficult transition for cancer patients between oncology and palliative care. A systematic review of decision-making for advanced cancer patients found that some oncologists continued treatment simply to maintain hope, rather than for clinical aims [146]. Discussions about side effects and fears of addiction can be challenging across all ethnic and racial groups, but as stated in one paper; they are nuanced by culture and ethnic background [106]. Clear culturally competent communication is important, particularly for people from minoritised groups [72]. Patient trust also plays a key role in challenging discussions. Patients from ethnically diverse groups may be less likely to have trust in the medical profession for historical reasons of discrimination and exploitation [147, 148]. Issues with trust have also been heightened recently during the COVID-19 pandemic [149], and even in 2017, a textbook for nurses containing culturally tailored information about pain was removed for using racist stereotypes [150].

Involving patient representatives from ethnically diverse backgrounds in designing medical education patient information and on pain management is important, both for building trust and developing culturally competent tools and approaches. Partnership working across different communities is also an essential part of taking anti-racist action within palliative care [138]. The findings on self-determination show people are not just passive consumers of medical advice, and are taking an active role in pain management.

### Public and Patient Involvement (PPI) representatives

PPI representatives shared their insights about the key findings from the review. They believed that those from ethnically diverse groups did suffer from more pain, and they all agreed that they still personally lived with pain on most days despite receiving care for pain. They reported it was difficult to make the doctors understand about the pain, and whilst they felt they had a good understanding of pain medication, they worried they were not necessarily getting the best medications available. PPI representatives pointed to systemic issues with getting doctor’s appointments, and one person experienced challenges accessing pain medication. Representatives from African and Caribbean heritage backgrounds shared evocative and emotional experiences of racial discrimination within UK healthcare, including during the recent COVID-19 pandemic. A South Asian patient representative felt that the representation of USA-based South Asians was similar to South Asians communities in the UK. Although one PPI representative emphasised the differences between USA and UK healthcare systems, and felt the UK should be understood differently.

### Limitations

The main limitation of this review was the focus on a small number of countries, and only including English language papers. The focus on English language was deliberate. The aim was to compare and contrast the minoritisation of different but recognisably similar ethnic groups in different countries, and to be able to include some Asian countries in this analysis. However, this was not successful. The inclusion of non-English language papers could have added richer evidence, in particular for the Asian countries (Singapore and Hong Kong). Building methodologically on a cross-national approach to include published papers in non-English language, is an important step to decolonise knowledge built and located through systematic reviewing. The limited scope produced a narrow evidence base: the majority of the literature was focused on cancer and in the USA. The focus on the USA means that there is a focus on a privatised model of healthcare and the findings may not be relevant for socialised national models of healthcare.

The primary outcome measure looked at differences in medication in standard care, not as part of research. Publication and outcome reporting bias are more difficult to assess in observational studies, as protocols are more rarely published on publicly available databases. One potential source of outcome reporting bias, particularly for the USA studies in the standardised collection of racial data. Racial data is more commonly collected in the USA in healthcare and other public institutions compared to other countries [151]. As this data is more readily available, studies which did not originally intend to investigate racial differences may choose to report them if they find strong correlations or unusual results. Likewise there may be many published observational studies of advanced disease in the USA with available racial data, but it has not been published because the findings do not show any strong trends. For the primary outcome measure, studies which did not contain racial or ethnic data on pain medication differences were excluded from the analysis, prospective protocols were not available to check these against. The overall risk of bias across the dataset was medium, 22/46 (48%) studies were rated as ‘Medium’ WoE [16, 95, 97, 99–114, 117, 129].

There is a need for better ethnicity data in other countries outside of USA, but with the caution of always considering data as socially and historically situated. A focus on individual level data only, which ignores the structural barriers many people face, could lead to essentialising and normalising pain management problems for people from ethnically diverse backgrounds, rather than tackling the problems. As recent events such as COVID-19 inequalities [30, 31, 33], Empire Windrush [29] and the Black Lives Matter campaign [28] have highlighted, data is powerful but it must understood in context.

## Conclusion

Overall there was not enough high quality quantitative evidence to draw a conclusion on the differences in receiving pain medication for people with advanced disease of different racial and ethnic groups. However, the mixed methods and qualitative evidence revealed a more complex picture. There were commonalities in fears and anxieties about pain medication across diverse ethnic groups; such as fears about side effects, tolerance and addiction. It also indicates that these fears and anxieties may be differently prioritised, and have differing foundations, which may be related to culture, faith, educational and social factors. The mixed methods analysis also showed some limited evidence on: differences in service provision and pain outcomes for people from ethnically diverse groups; difficulties with communication, and limited evidence on racial and ethnic stereotyping and the racial/ethnic dynamic within healthcare. These topics need to be explored further.

Despite the conceptual difficulties of researching and measuring race, ethnicity and pain management, it is important to note that the evidence brings into sharp relief the significant material differences in the reality of pain for many people with advanced disease. There is still a significant amount of undertreatment for pain across different conditions for many people from different ethnic groups. It is also indicative that those from racial and ethnically diverse groups may face other barriers to pain management. Pain remains a fundamental human rights issue which must be addressed [7, 8]. The COVID-19 pandemic has shown that global health emergencies widen and amplify health inequalities [30, 32, 33]. In addition the pandemic has reduced access to pain management services, [152] delayed testing and treatment for many advanced diseases including cancer [153] making these at risk populations even more vulnerable.

### Research and policy recommendations

To move towards a more equitable treatment of pain, we have the following recommendations for researcher and policy-makers:

- *Greater policy and research engagement with issues of ethnicity, race and racism within palliative care.* There is a dearth of pain management research that is inclusive of people from minoritised racial and ethnic groups. Future research in pain management should be address issues of ethnicity, race and racism, and should be inclusive from the conception and funding stages. Two recent editorials have made recommendations about how to achieve greater inclusivity within palliative care research [154, 155]. These include addressing race and ethnicity in every manuscript, naming structural racism and viewing genetic arguments about race with scepticism [154].
- *Research on pain management, race and ethnicity based outside of the USA*. The evidence base was heavily focused on the USA, and thus may have limited validity. Research needs to be replicated in other countries to determine the relevance and transferability of findings to other racial and ethnic communities in countries which have different cultural values and healthcare systems.
- *Community partnership working with racially and ethnically diverse groups to develop culturally competent pain management.* The evidence indicates issues with communication between doctor and patient, and high levels of unmet pain management need across many different ethnic groups. Partnership working is an important part of taking anti-racist action within palliative care [138]. It is crucial for creating approaches to challenging conversations about pain management that are ground-up, and centred around different diverse perspectives.

## Supplementary Information

**Additional file 1.**

## Data Availability

On request to corresponding author.

## Abbreviations

AMED: Allied and Complementary Medicine Database
BLM: Black Lives Matter
BPI: Brief Pain Inventory
CINAHL: Cumulative Index to Nursing and Allied Health Literature
COPD: Chronic Obstructive Pulmonary Disease
EDU: Equivalent Dose Units
EMBASE: Excerpta Medica database
EOL: End of Life
EThOS: Electronic Theses Online Service
HDI: Human Development Index
JBI: Joanna Briggs Institute
MEDD: Morphine Equivalent Daily Dose
PMI: Pain Management Index
PPI: Public and Patient Involvement
PRISMA: Preferred Reporting Items for Systematic reviews and Meta-Analyses
PROMs: Patient Reported Outcome Measures
RCT: Randomised Control Trial
UK: United Kingdom
UN: United Nations
USA: United States of America
WHO: World Health Organization
WoE: Weight of Evidence
