# Supplementary material for: Does ethnicity affect pain management for people with advanced disease? A mixed methods cross-national systematic review of ‘very high’ Human Development Index English-speaking countries
Source: BMC Palliat Care. 2022 Apr 6;21:46. doi: 10.1186/s12904-022-00923-6 (PMC8983802; doi:10.1186/s12904-022-00923-6)
Supplement: Supplementary file 1 — Additional file 1. [file 12904_2022_923_MOESM1_ESM.pdf]

Supplementary file appendices for: ‘Does ethnicity affect pain management for people with advanced disease? A mixed methods cross-national systematic review of ‘very high’ Human Development Index English-speaking countries’

Supplementary Table 1. Cross-tabulation of significant differences in receiving pain medication as part of standard care by race/ethnicity (N=18). Measured by: (a) standardised tools; (b) study designed measures

**First author Year Country *WoE*[1]**	**Patient population**	**Measurement tool or design[3]**	**Statistical Analysis**	**Analysis results by racial and ethnic groups[2]**	**(P-Value) Significance***	**Written summary**	**Significant results Y/N/M[4]**
(a) Standardised tool for pain management measurement							
1. Anderson<br>2000<br>USA<br>*High*	Cancer patients (108)	PMI	Descriptive comparative: percentage difference	**African American** / **Hispanic**<br>***% Negative PMI*** / ***% Negative PMI***<br>31% / 28%	(P value not reported)	No significant difference between African American and Hispanic patients in negative PMI.	**N**
2. Anderson<br>2002<br>USA<br>*High*	Cancer patients (31)	PMI	Descriptive comparative: Percentage difference	**African American** / **Hispanic**<br>***% Negative PMI*** / ***% Negative PMI***<br>36% / 35%	(P value not reported)	No significant difference between African American and Hispanic patients in negative PMI.	**N**
3. Fisch<br>2012<br>USA<br>*Medium*	Cancer patients (2026)	PMI<br><br>Dichotomous variable of based on PMI score.	Univariable and multivariable logistic regression	**White and non-Hispanic** / **Minority**<br>***No. (%)*** / ***No. (%)***<br>411 (29) / 221 (51)<br>*Undertreatment Initial*<br>*Univariate OR, (CI)* 0.38* (0.24-0.61) / *Univariate* 1.00 REF<br>*Multivariate OR, (CI)* 0.51* (0.37- 0.70) / *Multivariate* 1.00 REF<br>***No. (%)*** / ***No. (%)***<br>371 (30) / 180 (51)<br>*Undertreatment Follow-up*<br>*Univariate OR, (CI)* 0.41* (0.25-0.68) / *Univariate* 1.00 REF<br>*Multivariate OR, (CI)* 0.50* (0.35-0.70) / *Multivariate* 1.00 REF	(P=0.10)*<br>(P=0.002)*<br>(P=0.018)*<br>(P=0.001)*	White and Non-Hispanic patients were significantly less likely to experience undertreatment for pain compared to Minority patients at initial and follow-up stage.	**Y**

4. Hwang<br>2004<br>USA<br>*Medium*	Prostate cancer hospital patients (89)	MEDD<br><br>Proportions of patients using opioids and number of opioids used	Descriptive statistics: Percentage difference	<u>**Caucasian**</u> | <u>**African American**</u><br>% Using opioids: ***N (%)*** 44 (88) | ***N (%)*** 33 (89)<br>Number of opioids: ***Median (range)*** 2 (0-4) | ***Median (range)*** 2 (0-4)	(P=0.87)<br><br>(P=0.49)	No significant differences between the proportion of Caucasian and African American patients using opioids, and the number of opioids used.	**N**
5. Mosher<br>2010<br>USA<br>*Medium*	Cancer patients (87)	PMI<br>Score of 0 or above indicating adequate management	Univariate analyses of variance	<u>**African American**</u> | <u>**Spanish Speaking Latina**</u> | <u>**English Speaking Latina**</u> | <u>**Caucasian**</u> | **Multi-variate F**<br>*Score (SD)* | *Score (SD)* | *Score (SD)* | *Score (SD)*<br>- 0.53 (0.52) | - 0.78 (0.42) | - 0.80 (0.42) | - 0.56 (0.51) | 1.66	Not significant at P <0 .05 level<br><br>(P value not stated)	No significant differences in adequate pain management between the different groups.	**N**
6. Monroe<br>2010<br>USA<br>*Lower*	Nursing home dementia patients (55)	EDU	Descriptive comparative: Mean difference	<u>**African American**</u> *Mean (SD, SEM)* | <u>**Caucasian**</u> *Mean (SD, SEM)*<br>*EDU* 9.12 (11.81, 2.95) | *EDU* 9.8 (17.67, 2.73)	Not Significant at P<0.01 level<br><br>(P values not reported)	There was no significant difference) between African Americans and Caucasians on the Equivalent Dose Unit (EDU) scale.	**N**
(b) Within study designed measure of pain management							
7. Lamba<br>2020<br>USA<br>*High*	Patients with Brain Metastases (17,957)	Receiving non-opioids and opioids	Descriptive percentage comparison and Multivariate logistic regression	<u>**White**</u> | <u>**African American**</u> | <u>**Hispanic**</u> | <u>**Asian**</u><br>***No. (%)*** | ***No. (%)*** | ***No. (%)*** | ***No. (%)***<br>Prevalence Non-opioid: 615 (5) | 93 (5) | 40 (3) | 81 (7)<br>Prevalence Opioid: 6237 (46) | 1091 (53) | 601 (51) | 464 (38)<br>***OR, (CI), P*** | ***OR, (CI), P*** | ***OR, (CI), P*** | ***OR, (CI), P***<br>Regression Non-opioid: REF | 0.89 (0.66-1.20) P=0.44 | 0.69 (0.46-1.05) P=0.08 | 0.97 (0.70-1.34) P=0.86<br>Regression Opioid: REF | 0.96 (0.85-1.08) P=0.48 | 1.09 (0.94-1.26) P=0.24 | 0.86 (0.75-0.99) P=0.04*	P=0.001*<br>P<0.001*<br><br>Asian (P=0.04)*	There were significant differences in prevalence of opioids and non-opioids across the different ethnic groups. The regression analysis revealed Asian patients were less likely to receive opioids.	**Y**

8. Cea<br>2016<br>USA<br>*Medium*	Hospice discharge and home hospice patients (3,918)	Receiving opioid analgesics	Multivariate logistic regression	**Non-Hispanic White**: REF, N/A<br>**Non-Hispanic Black** AOR (CI): 0.82 (0.59-1.14)<br>**Hispanic** AOR (CI): 0.62* (0.40-0.97)<br>**Other** AOR (CI): 0.96 (0.48-1.92)	*Significant at P<0.05 level<br>(P value not reported)	Compared to Non-Hispanic White patients, Hispanic patients were significantly less likely to receive opioid analgesics.	**Y**
9. Check<br>2016<br>USA<br>*Medium*	Breast cancer patients (883)	Use of opioid analgesic	Modified Poisson regression	**White** *Risk (95% CI)* / **Black** *Risk (95% CI)* / **White vs Black** *Risk Ratio (95% CI)*<br>*Unadjusted*: 0.61 (0.57-0.64) / 0.60 (0.36-0.56) / 0.98 (0.84-1.14)<br>Adjusted: 0.45 (0.36-0.56) / 0.47 (0.38-0.57) / 0.97 (0.84- 1.13)	(P values not reported)	There were no significant differences in opioid use between White and Black patients.	**N**
10. Fischer<br>2007<br>USA<br>*Medium*	Cancer patients in veterans' hospital (217)	Treatment of pain, if present	Logistic regression	**White** *REF*: 1<br>**African American** *OR (95% CI)*: 0.69 (0.191-2.48)<br>**Hispanic White** *OR (95% CI)*: 0.51 (0.14-1.86)	(No P Value reported)	There were no significant differences between White, African American and Hispanic White patients for treatment of pain.	**N**
11. Gerlach<br>2021<br>USA<br>*Medium*	Medicare Hospice beneficiary (554,022)	Receiving opioids	Multivariate logistic regression	**Non-Hispanic White**: REF 1<br>**Non-Hispanic Black** ***AOR (CI)***: 0.75* (0.72,0.77)<br>**Hispanic** ***AOR (CI)***: 0.74* (0.70,0.78)<br>**Other** ***AOR (CI)***: 0.84* (0.80,0.87)	*Significant at P<0.001 level (P Values not reported)	Compared to white Medicare hospice beneficiaries, non-Hispanic black beneficiaries Were significantly less likely to receive opioids	**Y**
12. Gurney<br>2021<br>New Zealand<br>*Medium*	Lung cancer patients (20,081)	Accessing opioid and non-opioid analgesics	Logistic regression	**Māori** ***Adj. %, Adj OR (CI)*** / **non-Māori** ***Adj. %, Adj OR (CI)***<br>Any pain medication: 90, 1, (0.85-1.19) / 89, REF<br>Non-opioid: 78, 0.89 (0.79-1.01) / 78, REF<br>Mild opioid: 41, 1 (0.9-1.12) / 41, REF	(No P Value reported)	There were no significant differences in using opioid and non-opioid medication between patients from Māori and non-Māori ethnic groups.	**N**

				Strong opioid: 78, 1.08 (0.96-1.23); 76, REF			
13. Pinheiro<br>2019<br>USA<br>*Medium*	Breast cancer patients (23,091)	Proportions of patients using opioids	Modified Poisson model	**Non-Hispanic**: **US-born**, **Foreign-born**; **Hispanic**: **US-born**, **Foreign born**<br><br>% Using opioid: ***N (%)*** US-born Non-Hispanic 14,097 (69%); Foreign-born Non-Hispanic 797 (62%); US-born Hispanic 709 (75%); Foreign born Hispanic 287 (60%)<br><br>Opioid Risk ratio: US-born Non-Hispanic ***REF*** N/A; Foreign-born Non-Hispanic ***RR, (CI), P*** 0.91 (0.87-0.95) P<0.001*; US-born Hispanic ***RR, (CI), P*** 1.06 (1.02-1.10) P<0.01*; Foreign born Hispanic ***RR, (CI), P*** 0.86 (0.80-0.92) P<0.001*	(P < 0.0001)*<br><br>(P<0.001*)<br>(P<0.01*)<br>(P<0.001*)	Compared to US-born non-Hispanic women, foreign-born women, and US born Hispanic women used significantly fewer opioids, even when adjusting for demographic, tumour and treatment.	**Y**
14. Reynolds<br>2008<br>USA<br>*Medium*	Nursing home residents with pain (1,133)	Proportion with documented pain medication	Descriptive comparative: Percentage difference	**White** ***No (%)***; **Minority** ***No (%)***<br><br>Pain med (non PRN acetaminophen): 862 (52.6); 262 (50.0)<br><br>Scheduled medication: 862 (33.6); 262 (34.5)	(P=0.558)<br><br>(P=0.507)	There were no significant differences between white and minority patients' pain treatment use.	**N**
15. Halpern<br>2019<br>USA<br>*Lower*	Breast and colorectal cancer patients (8,438)	Receiving pain management from Medicaid data	Modified Poisson regression model	Breast cancer patients: **Non-Hispanic Black**; **Hispanic**; **All other race**<br>*REF Non-Hispanic White*: ***RR (P Value)*** 0.56 (P=0.0005)*; ***RR (P Value)*** 0.41 (P<0.0001)*; ***RR (P Value)*** 0.58 (P=0.0079)*<br><br>Colorectal cancer patients: **Non-Hispanic Black**; **Hispanic**; **All other race**<br>*REF Non-Hispanic White*: ***RR (P Value)*** 0.62 (Not stated); ***RR (P Value)*** 1.63 (Not stated); ***RR (P Value)*** 0.85 (Not stated)	(P=0.0005)*<br>(P<0.0001)*<br>(P=0.0079)*<br><br>Not significant at (P>0.05)	Compared with Non-Hispanic White breast cancer patients, patients from Non-Hispanic Black patients, Hispanic patients and patients from all other race groups had a decreased likelihood of receiving pain medication.<br><br>There were no significant differences in receiving pain medication by racial groups for colorectal cancer patients.	**M**

16. Rolnick<br>2007<br>USA<br>*Lower*	Ovarian cancer patients (421)	Receiving high intensity pain medication	Multivariate logistic regression	**White** *OR, (CI)*<br>Receiving high intensity pain medication: 1.419 (0.812- 2.482)	Results not significant at P<0.05<br><br>P=0.219	No statistically significant difference was found by race.	**N**
17. Saphire<br>2020<br>USA<br>*Lower*	Lung cancer decedents (16,246)	Receiving pain medication	Multivariate logistic regression	*Pain medication*: REF White Non-Hispanic<br>**Black Non-Hispanic** *RR, (P Value), CI*: 0.79 (P=0.001)* 0.69-0.91<br>**Hispanic** *RR, (P Value), CI*: 0.74 (P<0.001)* 0.63-0.87<br>**Asian, Other** *RR, (P Value), CI*: 0.57 (P<0.001)* 0.49-0.65	(P=0.001)*<br>(P<0.001)*<br>(P<0.001)*	Compared to Non-Hispanic White decedents, Non-Hispanic Black, Hispanic and Asian/Other decedents were significantly less likely to receive pain medication.	**Y**
18. Wieder<br>2014<br>USA<br>*Lower*	Patients with cancer pain (360)	Use of long acting opioids	Descriptive statistics: comparison, and logistic regression	**African American** *No.*: Yes 84; No 87<br>**Hispanic** *No.*: Yes 45; No 75<br>**Caucasian** *No.*: Yes 20; No 25<br>**Asian** *No.*: Yes 3; No 15<br>**Not specified** *No.*: Yes 1; No 5<br><br>Logistic regression:<br>**Race: Asian or Hispanic** **OR, (P Value)**: 0.606 (P=0.158)<br>**Race: Caucasian** *OR (P Value)*: 1.007 (P=0.987)	(P=0.027)*<br><br>P-level not stated	Hispanic and Asian patients were prescribed long acting opiates at a lower rate than expected by the distribution of use in the entire sample, but ethnicity was not a predictor in the regression analysis.	**M**

Supplementary Table 1.Footnotes

1. Gough's Weigh of Evidence Framework (WoE). Category D – total score.
2. Racial and ethnic groups as described within the original research papers.
3. Pain management tools. **PMI** = Pain Management Index. **EDU** = Equivalent Dose Units. **MEDD** = Morphine Equivalent Daily Dose
4. Did the study have significant results for ethnicity and pain level difference? **Y** = Yes. **N** = No. **M** = Mixed.

* = Significant result. Significance levels set within each individual study.

Supplementary Table 2. Thematic and conceptual matrix of themes from the included studies in the mixed methods analysis (N=46)

**Structural levels of healthcare**	**Grouped Themes 1 - Patient and family perspectives**	**Grouped Themes 2- Barriers to pain management**	**Grouped Themes 3 - Service level and structural issues**
Personal concerns and individual needs<br><br>Healthcare service and health care providers<br><br>Wider society, research and broader healthcare services issues	Fears and concerns<br><br>Self-determination<br>Unmet pain management needs	Information and misconceptions<br>Racial/ethnic interactions and dynamics within healthcare<br>Doctor-patient communication<br><br>Racial and ethnic stereotyping	Pain outcomes following treatment<br>Differences in the utilisation of standard pain management care<br>Research based studies

Supplementary Table 3. Search strategies

Journal searches	
**Search used for Medline, AMED, PsychInfo and EMBASE**<br>Limits: English language only<br>Year 2000 to current (24th Aug 2021)	1. "advanced disease".ab,kf,ti.<br>2. metastatic.ab,kf,ti.<br>3. "progressive disease".ab,kf,ti.<br>4. palliative.ab,kf,ti.<br>5. "terminal*".ab,kf,ti.<br>6. hospice.ab,kf,ti.<br>7. "life limiting".ab,kf,ti.<br>8. "end stage".ab,kf,ti.<br>9. "progressive neurological disease".ab,kf,ti.<br>10. "futil*".ab,kf,ti.<br>11. "end of life".ab,kf,ti.<br>12. hospices.mp. or exp Hospices/<br>13. hospice care.mp. or exp Hospice Care/<br>14. palliative care.mp. or exp Palliative Care/<br>15. terminal care.mp. or exp Terminal Care/<br>16. 1 or 2 or 3 or 4 or 5 or 6 or 7 or 8 or 9 or 10 or 11 or 12 or 13 or 14 or 15<br>17. pain.ab,kf,ti.<br>18. "analgesi*".ab,kf,ti.<br>19. opioid.ab,kf,ti.<br>20. morphine.ab,kf,ti.<br>21. codeine.ab,kf,ti.<br>22. fentanyl.ab,kf,ti.<br>23. hydrocodone.ab,kf,ti.<br>24. hydromorphone.ab,kf,ti.<br>25. tramadol.ab,kf,ti.<br>26. oxycodone.ab,kf,ti.<br>27. meperidine.ab,kf,ti.<br>28. TENS.ab,kf,ti.<br>29. "Transcutaneous Electric Nerve Stimulation".ab,kf,ti.<br>30. lidocaine.ab,kf,ti.<br>31. prilocaine.ab,kf,ti.

	32. "Non-steroidal anti-inflammatory drug* ".ab,kf,ti.<br>33. "nsaid*".ab,kf,ti.<br>34. pregabalin.ab,kf,ti.<br>35. gabapentin.ab,kf,ti.<br>36. ketamine.ab,kf,ti.<br>37. Physiotherapy.ab,kf,ti.<br>38. diphosphonates.ab,kf,ti.<br>39. "biphosphonates".ab,kf,ti.<br>40. acetaminophen.ab,kf,ti.<br>41. Corticosteroids.ab,kf,ti.<br>42. exp Analgesia/ or analgesia.mp.<br>43. opioid.mp. or exp Analgesics, Opioid/<br>44. exp Pain/an, co, di, dg, de, dt, ph, pp, pc, px, st [Analysis, Complications, Diagnosis, Diagnostic Imaging, Drug Effects, Drug Therapy, Physiology, Physiopathology, Prevention & Control, Psychology, Standards]<br>45. 17 or 18 or 19 or 20 or 21 or 22 or 23 or 24 or 25 or 26 or 27 or 28 or 29 or 30 or 31 or 32 or 33 or 34 or 35 or 36 or 37 or 38 or 39 or 40 or 41 or 42 or 43 or 44<br>46. ethnicity.ab,kf,ti.<br>47. ethnic.ab,kf,ti.<br>48. race.ab,kf,ti.<br>49. racial.ab,kf,ti.<br>50. multicultural.ab,kf,ti.<br>51. BAME.ab,kf,ti.<br>52. "black asian minority ethnic".ab,kf,ti.<br>53. "person of color".ab,kf,ti.<br>54. "person of colour".ab,kf,ti.<br>55. "aborigin*".ab,kf,ti.<br>56. ethnocultural.ab,kf,ti.<br>57. heritage.ab,kf,ti.<br>58. exp Culture/<br>59. ethnic groups.mp. or exp Ethnic Groups/<br>60. continental population groups.mp. or exp Continental Population Groups/<br>61. 46 or 47 or 48 or 49 or 50 or 51 or 52 or 53 or 54 or 55 or 56 or 57 or 58 or 59 or 60<br>62. 16 and 45 and 61

**CINAHL**<br>Limits: English language. 2000 to 24th Aug 2021	AB "advanced disease" OR AB metastatic OR AB "progressive disease" OR AB terminal OR AB hospice OR AB "life limiting" OR AB "end stage" OR AB "progressive neurological disease" OR AB "futile treatment" OR AB "end of life" OR palliative<br>AND<br>AB pain OR AB analgesi* OR AB opioid epidemic OR AB morphine OR AB codeine OR AB fentanyl OR AB hydrocodone OR AB hydromorphone OR AB tramadol OR AB oxycodone OR AB meperidine OR AB TENS OR AB "Transcutaneous Electric Nerve Stimulation" OR AB lidocaine OR AB prilocaine OR AB (non-steroidal anti-inflammatory drugs or nsaids) OR AB corticosteroids OR AB pregabalin OR AB gabapentin OR AB ketamine OR AB physiotherapy OR AB disphosphonates OR AB biphosphonates OR AB acetaminophen<br>OR<br>AB ethnicity OR AB ethnic* OR AB race OR AB racial OR AB multicultural OR AB bame OR AB "black asian minority ethnic" OR AB "person of color" OR AB "person of colour" OR AB aborig* OR AB ethnocultural OR AB heritage
**Grey literature**	
**ETHoS**<br>No full Boolean search available. Instead the following terms were used, and the titles of duplicates or those before the year 2000 were not downloaded.	Palliative and ethnicity<br>Palliative and race<br>Palliative and BIPOC<br>Palliative and BAME<br>Palliative and heritage<br>palliative AND "person of colour"<br>palliative AND "person of color"<br>Hospice and ethnicity<br>Hospice and race<br>Hospice and BIPOC<br>Hospice and BAME<br>Hospice and heritage<br>Hospice AND "person of colour"<br>Hospice AND "person of color"<br>“advanced disease” and ethnicity<br>“advanced disease” and race<br>“advanced disease” and BIPOC<br>“advanced disease” and BAME

	“advanced disease” and heritage<br>“advanced disease” AND "person of colour"<br>“advanced disease” AND "person of color"<br>Pain and ethnicity<br>Pain and race<br>Pain and BIPOC<br>Pain and BAME<br>Pain and heritage<br>Pain AND "person of colour"<br>Pain AND "person of color"
**Journals**<br>Palliative medicine<br>BMJ Supportive and Palliative Care<br>European Journal of Palliative Care<br>BMC Palliative Care<br>Ethnicity and Healthcare<br>Ethnic and racial studies	Search of titles last 5 years up to 24th August 2021
**Grey literature and websites**<br>Marie Curie online<br>Macmillan online<br>NICE evidence search<br>OpenGrey	Search of pages all or last 5 years up to 24th August 2021 as relevant

PRISMA 2020 Checklist

Section and Topic	Item #	Checklist item	Location where item is reported
**TITLE**			
Title	1	Identify the report as a systematic review.	p.1
**ABSTRACT**			
Abstract	2	See the PRISMA 2020 for Abstracts checklist.	p.1
**INTRODUCTION**			
Rationale	3	Describe the rationale for the review in the context of existing knowledge.	p.4-5
Objectives	4	Provide an explicit statement of the objective(s) or question(s) the review addresses.	p.5
**METHODS**			
Eligibility criteria	5	Specify the inclusion and exclusion criteria for the review and how studies were grouped for the syntheses.	p.8
Information sources	6	Specify all databases, registers, websites, organisations, reference lists and other sources searched or consulted to identify studies. Specify the date when each source was last searched or consulted.	p.9
Search strategy	7	Present the full search strategies for all databases, registers and websites, including any filters and limits used.	Supplementary appendices
Selection process	8	Specify the methods used to decide whether a study met the inclusion criteria of the review, including how many reviewers screened each record and each report retrieved, whether they worked independently, and if applicable, details of automation tools used in the process.	p.10
Data collection process	9	Specify the methods used to collect data from reports, including how many reviewers collected data from each report, whether they worked independently, any processes for obtaining or confirming data from study investigators, and if applicable, details of automation tools used in the process.	p.10
Data items	10a	List and define all outcomes for which data were sought. Specify whether all results that were compatible with each outcome domain in each study were sought (e.g. for all measures, time points, analyses), and if not, the methods used to decide which results to collect.	p.10
	10b	List and define all other variables for which data were sought (e.g. participant and intervention characteristics, funding sources). Describe any assumptions made about any missing or unclear information.	p.10
Study risk of bias assessment	11	Specify the methods used to assess risk of bias in the included studies, including details of the tool(s) used, how many reviewers assessed each study and whether they worked independently, and if applicable, details of automation tools used in the process.	p.11
Effect measures	12	Specify for each outcome the effect measure(s) (e.g. risk ratio, mean difference) used in the synthesis or presentation of results.	p.10
Synthesis methods	13a	Describe the processes used to decide which studies were eligible for each synthesis (e.g. tabulating the study intervention characteristics and comparing against the planned groups for each synthesis (item #5)).	p.11-12
	13b	Describe any methods required to prepare the data for presentation or synthesis, such as handling of missing summary statistics, or data conversions.	p.11-12
	13c	Describe any methods used to tabulate or visually display results of individual studies and syntheses.	Tabulate p.11-12<br>N/A Visualise
	13d	Describe any methods used to synthesize results and provide a rationale for the choice(s). If meta-analysis was performed, describe the	p.11-12

Section and Topic	Item #	Checklist item	Location where item is reported
		model(s), method(s) to identify the presence and extent of statistical heterogeneity, and software package(s) used.	
	13e	Describe any methods used to explore possible causes of heterogeneity among study results (e.g. subgroup analysis, meta-regression).	N/A
	13f	Describe any sensitivity analyses conducted to assess robustness of the synthesized results.	p.11
Reporting bias assessment	14	Describe any methods used to assess risk of bias due to missing results in a synthesis (arising from reporting biases).	p.11
Certainty assessment	15	Describe any methods used to assess certainty (or confidence) in the body of evidence for an outcome.	p.11
**RESULTS**			
Study selection	16a	Describe the results of the search and selection process, from the number of records identified in the search to the number of studies included in the review, ideally using a flow diagram.	p.13
	16b	Cite studies that might appear to meet the inclusion criteria, but which were excluded, and explain why they were excluded.	p.13
Study characteristics	17	Cite each included study and present its characteristics.	Table 2.
Risk of bias in studies	18	Present assessments of risk of bias for each included study.	Table 2.
Results of individual studies	19	For all outcomes, present, for each study: (a) summary statistics for each group (where appropriate) and (b) an effect estimate and its precision (e.g. confidence/credible interval), ideally using structured tables or plots.	Table 2 for overall summary.<br>Supplementary Table 1 for Primary outcome measure
Results of syntheses	20a	For each synthesis, briefly summarise the characteristics and risk of bias among contributing studies.	Primary outcome measure p.13-15
	20b	Present results of all statistical syntheses conducted. If meta-analysis was done, present for each the summary estimate and its precision (e.g. confidence/credible interval) and measures of statistical heterogeneity. If comparing groups, describe the direction of the effect.	N/A
	20c	Present results of all investigations of possible causes of heterogeneity among study results.	N/A
	20d	Present results of all sensitivity analyses conducted to assess the robustness of the synthesized results.	p.21
Reporting biases	21	Present assessments of risk of bias due to missing results (arising from reporting biases) for each synthesis assessed.	N/A Discussion on p.27
Certainty of evidence	22	Present assessments of certainty (or confidence) in the body of evidence for each outcome assessed.	p.15 and p.21
**DISCUSSION**			

Section and Topic	Item #	Checklist item	Location where item is reported
Discussion	23a	Provide a general interpretation of the results in the context of other evidence.	p.21-26
	23b	Discuss any limitations of the evidence included in the review.	p.26-27
	23c	Discuss any limitations of the review processes used.	p.26-27
	23d	Discuss implications of the results for practice, policy, and future research.	p.28-28
**OTHER INFORMATION**			
Registration and protocol	24a	Provide registration information for the review, including register name and registration number, or state that the review was not registered.	p.1
	24b	Indicate where the review protocol can be accessed, or state that a protocol was not prepared.	p.5
	24c	Describe and explain any amendments to information provided at registration or in the protocol.	p.5
Support	25	Describe sources of financial or non-financial support for the review, and the role of the funders or sponsors in the review.	p.31
Competing interests	26	Declare any competing interests of review authors.	p.31
Availability of data, code and other materials	27	Report which of the following are publicly available and where they can be found: template data collection forms; data extracted from included studies; data used for all analyses; analytic code; any other materials used in the review.	p.31

From: Page MJ, McKenzie JE, Bossuyt PM, Boutron I, Hoffmann TC, Mulrow CD, et al. The PRISMA 2020 statement: an updated guideline for reporting systematic reviews. BMJ 2021;372:n71. doi: 10.1136/bmj.n71

For more information, visit: http://www.prisma-statement.org/
